# Effects of standalone plyometric training on vertical jump and linear sprint performance in handball athletes: a systematic review and meta-analysis

**DOI:** 10.3389/fphys.2026.1863791

**Published:** 2026-06-30

**Authors:** Chaonan Zhang, Qiang Wang

**Affiliations:** 1 College of Physical Education, Kashi University, Kashgar Prefecture, Xinjiang Uygur Autonomous Region, China; 2 College of Physical Education, Kashi University, Kashi, China

**Keywords:** age moderation, handball, handball athletes, linear sprint, meta-analysis, standalone plyometric training, vertical jump

## Abstract

**Introduction:**

Handball is a high-intensity intermittent sport that demands exceptional lower-body explosive power for jumping and sprinting in match play. Existing research on plyometric training for handball athletes often combines this modality with other interventions, leaving the independent effects of standalone plyometric training on key physical outcomes poorly quantified. This meta-analysis aimed to systematically evaluate the efficacy of standalone plyometric training on vertical jump (squat jump [SJ], countermovement jump [CMJ]) and multi-distance linear sprint performance in handball athletes, and to explore moderators of training response.

**Methods:**

A systematic search was performed in PubMed, Web of Science, Embase, Cochrane Library, and SportDiscus up to March 2, 2026. Controlled trials comparing standalone plyometric training with a no-intervention control in handball athletes were included. Methodological quality was assessed via the ROBINS-I tool. Pooled Hedges’ g effect sizes with 95% confidence intervals (CI) were calculated using a random-effects model. Heterogeneity, publication bias, and subgroup effects (age, gender) were assessed via standard meta-analytic methods.

**Results:**

Fourteen studies with 350 handball athletes were included. Standalone plyometric training significantly improved both CMJ (g = 1.17, 95% CI 0.40–1.93, I² = 82.0%) and SJ (g = 0.86, 95% CI 0.33–1.39, I² = 64.7%) performance. No significant effects were observed for 10m, 20m, or 30m linear sprint outcomes. Subgroup analyses showed significant vertical jump benefits in athletes under 18 years, but not in adults aged 18 and above, with no significant gender difference in CMJ training response. Significant publication bias was identified for CMJ and 30m sprint outcomes, with borderline bias for SJ.

**Conclusion:**

Standalone plyometric training effectively improves vertical jump performance in handball athletes, particularly in adolescent players, but has no significant benefit for short-to-moderate distance linear sprint performance. Future research should establish standardized handball-specific plyometric protocols to refine evidence-based conditioning guidelines.

**Systematic Review Registration:**

https://www.crd.york.ac.uk/prospero/, identifier CRD420261360294.

## Introduction

Handball is a high-intensity intermittent team sport that requires athletes to repeatedly execute technical actions such as jump shots, breakthrough sprints, sudden stops, directional changes, and defensive blocking during offensive and defensive transitions, placing exceptional demands on lower-body explosive power and acceleration capabilities (Stojanović et al., 2024; Scanlan et al., 2021; Trajkovic et al., 2022). Among these physical attributes, lower-body explosive power represents a core determinant of handball-specific performance, directly impacting a player’s jump height for shooting, blocking capacity during defense, and starting acceleration ability in fast-break situations (Šarabon et al., 2021; Trajkovic et al., 2025). Given this close association with on-court match performance, the countermovement jump (CMJ, with pre-stretch buffering relying on the stretch-shortening cycle), squat jump (SJ, without pre-stretch, focusing on pure concentric contraction), and short-to-moderate distance linear sprints serve as classic and widely accepted tests for evaluating lower-body explosive power and acceleration in handball athletes. Specifically, SJ effectively assesses concentric-dominant explosive strength linked to handball-specific movements, such as immediate vertical takeoff for shooting without pre-squat in crowded goal areas, or sudden jump for loose ball contests in restricted spaces, which has been validated in prior handball literature (Mirkov et al., 2021). CMJ, by contrast, aligns more closely with the force production pattern of most jump shots and defensive blocking jumps in handball matches that involve pre-stretch actions, and is recognized as one of the gold standards for evaluating specific explosive power in handball athletes (Aloui et al., 2020; Kozinc et al., 2024). Meanwhile, 10m, 20m, and 30m linear sprint performance directly corresponds to the linear acceleration ability required for fast-break breakthroughs and defensive backtracking in handball, and is a key physical indicator distinguishing handball athletes of different competitive levels (Milanović et al., 2023).

To enhance these critical athletic qualities, plyometric training has become a fundamental component of modern handball strength and conditioning regimens (Falch et al., 2022). Grounded in the exploitation of the stretch-shortening cycle (SSC), plyometric exercises consisting of various depth jumps, bounds, hops, and jump variations are designed to elicit rapid, high-power muscular contractions (Komi, 2000; Behm and Sale, 2021). The underlying physiological mechanism involves a pre-stretch of the muscle-tendon unit that stores elastic energy, thereby facilitating amplified force output during the subsequent concentric contraction (Komi, 2000). Long-term plyometric training induces robust neuromuscular adaptations, including enhanced stretch reflex sensitivity, optimized motor unit recruitment patterns, increased rate of force development (RFD), and improved musculotendinous stiffness, all of which collectively improve lower-body explosive power performance (Taube et al., 2012).

A substantial body of primary research has examined the effects of plyometric training on handball performance. For instance, numerous studies—including those by Chelly et al. (2014); Chaabene et al. (2019), and Gaamouri et al. (2023)—have specifically investigated its impact on key physical performance metrics in handball athletes. These include significant improvements in vertical jump height, encompassing both CMJ and SJ performance, in junior handball players following a structured plyometric intervention (Hammami et al., 2022b). Marked enhancements in SJ capacity have also been documented in adolescent male handball athletes (Hermassi et al., 2023). For young female handball athletes, plyometric training has been shown to improve sprint acceleration and explosive power (Attene et al., 2015; Noutsos et al., 2021). Additionally, positive changes in linear sprint and change-of-direction ability have been reported following complex training interventions that incorporate plyometric exercises (Cavaco et al., 2014). Collectively, this primary literature forms a foundational evidence base exploring the efficacy of plyometric exercises for handball-specific athletic development. However, existing evidence syntheses are limited in several key aspects. First, they often confound the unique, independent contribution of plyometrics by combining this training modality with other interventions (e.g., resistance training, technical training), leaving the isolated effects of standalone plyometric training on key physical outcomes in handball athletes poorly quantified (Bouteraa et al., 2020; Rønnestad et al., 2021; Ramirez-Campillo et al., 2022, 2024; de Villarreal et al., 2024; Ellefsen et al., 2025). Second, many existing summaries rely on qualitative narrative reviews rather than comprehensive meta-analyses, thus failing to provide pooled, quantitative effect estimates of the intervention, and have not systematically explored key moderators of training response such as age and gender (Aloui et al., 2021; Moran et al., 2019; Falch et al., 2023). Building upon this foundation, this systematic review and meta-analysis quantitatively synthesizes evidence for the effects of standalone plyometric training on CMJ, SJ, and multi-distance (10m, 20m, 30m) linear sprint performance in handball athletes, and explores moderators of training response, thereby establishing an evidence-based foundation for optimizing handball-specific plyometric training prescriptions.

## Methods

This review was performed in accordance with the PRISMA statement (Page et al., 2021), and the protocol was prospectively registered in the PROSPERO database (CRD420261360294).

### Search strategy and selection

A systematic electronic literature search was implemented across five authoritative academic databases, namely PubMed, Web of Science, Embase, the Cochrane Library, and SportDiscus. The retrieval scope covered all peer-reviewed publications indexed in each database from its establishment to March 2, 2026. The core dimensions of the retrieval strategy included terminology related to isolated plyometric training (e.g., “plyometric intervention”, “jump training”, “explosive power training”), handball participants (including “handball”, “handball player”, “handball athlete*”), and corresponding physical performance outcomes, namely countermovement jump, squat jump, and linear sprint performance over multiple distances. These core dimensions were connected via Boolean operators (“AND” and “OR”), and standardized controlled vocabulary terms (such as MeSH terms in the PubMed database) were applied in applicable databases. The full retrieval strategy for each database is presented in **Supplementary File 1**.

After eliminating duplicate records, the titles and abstracts of the remaining citations underwent a preliminary assessment for relevance by two independent reviewers (C.Z. and Q.W.). The full texts of studies deemed potentially eligible were then retrieved and evaluated against the predefined inclusion criteria. Any discrepancies between the two reviewers during the entire selection process were resolved through consensus discussion; any persisting disagreements were adjudicated by C.Z. (the senior reviewer).

### Eligibility criteria

Eligibility of studies was assessed against the following pre-specified criteria: (a) the study participants were competitive handball athletes, with no limitations placed on age, sex, or competitive grade; (b) the experimental intervention was a standardized isolated plyometric training program, with no supplementary training interventions administered to the intervention cohort; (c) a control arm was included, which either received no targeted plyometric training or maintained their usual routine training without modification; (d) the study reported quantitative outcome data for at least one of the following physical performance metrics: countermovement jump (CMJ) height, squat jump (SJ) height, 10-meter linear sprint time, 20-meter linear sprint time, or 30-meter linear sprint time.

Eligible study designs were limited to randomized controlled trials and quasi-experimental controlled studies, both of which must contain an intervention group and a parallel control condition. In addition, studies eligible for inclusion must provide complete post-intervention statistical data, specifically the mean values, standard deviations, and sample sizes of both the intervention and control groups, to enable the calculation of Hedges’ g effect sizes.

Studies were excluded from the meta-analysis if they met any of the following criteria: the study participants were not confirmed as competitive handball athletes; the study design did not include a control or comparison arm; the intervention delivered was not isolated plyometric training as the core content, or combined plyometric training with other additional training modalities; the study failed to report the core outcome indicators of interest in this meta-analysis (countermovement jump, squat jump, 10-m linear sprint, 20-m linear sprint, or 30-m linear sprint); or the available statistical data were incomplete and could not support the calculation of effect sizes.

### Data extraction

A standardized data extraction protocol was established to guarantee the systematic and comprehensive collection of relevant information from all eligible studies. The extraction process was independently performed by two reviewers (C.Z. and Q.W.) using a pre-piloted standardized data extraction form. The following data items were systematically extracted and documented: first author’s surname, year of publication, country where the study was carried out, specific research design, participant demographic characteristics (including total sample size, age group, mean age, sex distribution, and competitive level), intervention-related parameters (encompassing standalone plyometric training modality, weekly session frequency, total intervention duration, training volume per session, and implementation context), outcome measurements (specifically countermovement jump height, squat jump height, 10-meter linear sprint time, 20-meter linear sprint time, and 30-meter linear sprint time), as well as potential moderating variables (such as age category, gender, total intervention length, and overall training volume).

For the purpose of quantitative synthesis, baseline and post-intervention data were extracted for both the experimental and control groups, including mean values, corresponding standard deviations, and sample sizes for all primary outcomes. In cases where studies directly reported change scores (computed as post-intervention values minus baseline values), these data were directly adopted for the meta-analysis. Where change scores were not provided but sufficient raw baseline and post-intervention data were available, the change scores were calculated manually.

Consistency between the two data extractors was verified through duplicate cross-checking procedures. Any unresolved discrepancies that persisted after cross-checking were resolved via consultation with a senior methodological reviewer (C.Z.) to reach a final binding decision.

### Risk of bias

A standardized risk of bias assessment procedure was implemented to ensure systematic and consistent evaluation of the methodological quality for all eligible studies. The assessment process was conducted independently by two reviewers (C.Z. and Q.W.) using the validated Risk of Bias in Non-randomized Studies of Interventions (ROBINS-I) tool (Sterne et al., 2016). The following core bias domains were systematically assessed and documented for each included study: confounding, selection of participants, classification of interventions, deviations from intended interventions, missing data, measurement of outcomes, and selection of the reported result. Each domain was assigned a risk rating of low, moderate, serious, or critical bias, and an overall risk of bias judgement was determined for each study based on the domain-level assessments.

For methodological quality synthesis, domain-specific risk ratings and overall risk of bias judgements were collated for both randomized and non-randomized controlled studies included in the meta-analysis. When studies provided complete and transparent methodological details to support a clear risk judgement, the corresponding rating was directly adopted for the final synthesis. In cases where studies reported insufficient methodological information to inform a definitive rating, a full-text re-examination was performed, and a conservative risk rating was assigned in line with ROBINS-I reporting guidelines (Sterne et al., 2016).

Consistency among the two independent assessors was ensured through iterative cross-checking of all domain ratings and overall risk judgements, and any persisting discrepancies were resolved through consultation with a senior methodological researcher (C.Z.) for final determination.

### Publication bias and sensitivity analysis

Potential publication bias for each primary outcome (countermovement jump, squat jump, 10m, 20m, and 30m linear sprint) was evaluated through a combination of visual inspection of funnel plot symmetry and quantitative statistical testing using Egger’s regression method (Egger et al., 1997). Funnel plot asymmetry was interpreted as non-significant when the associated two-tailed p-value exceeded 0.05, while a p-value below 0.05 was deemed indicative of statistically significant publication bias. To test the stability and reliability of the pooled meta-analytic findings, sensitivity analyses were implemented using a predefined two-stage approach: initially by removing studies judged to have serious or critical overall risk of bias via the ROBINS-I tool, and subsequently by systematically excluding each individual study in turn according to the leave-one-out procedure (Viechtbauer and Cheung, 2010). All analytical methods were aligned with the standards and guidance outlined in the Cochrane Handbook for Systematic Reviews of Interventions (Higgins et al., 2024).

### Statistical analysis

A series of meta-analyses were performed to quantify the effects of standalone plyometric training on core physical performance outcomes in handball athletes, utilizing pre-post change differences (calculated as post-intervention minus pre-intervention values) from both the standalone plyometric training intervention groups and no-intervention control groups. Pooled treatment effects were expressed as Hedges’ g with corresponding 95% confidence intervals (CIs) to correct for potential small-sample bias (Hedges and Olkin, 1985). All analyses adopted a random-effects model to accommodate the anticipated clinical and methodological variations across the included studies. Between-study variance was estimated via the restricted maximum likelihood method, and the Hartung-Knapp adjustment was applied for 95% CI computation to improve the reliability and stability of the pooled results.

Heterogeneity across included studies was assessed using the I² statistic, with values of 25%, 50%, and 75% deemed to represent low, moderate, and substantial between-study heterogeneity, respectively (Higgins et al., 2003). For outcomes presenting with substantial heterogeneity (I² > 50%), predefined subgroup analyses based on age group (<18 years vs. ≥18 years) and gender (male vs. female) were conducted to explore potential sources of between-study variation. Publication bias for each primary outcome was evaluated visually using funnel plots and statistically via Egger’s linear regression test (Egger et al., 1997). All statistical calculations were performed using R statistical software (version 4.4.1) with the metafor package (Viechtbauer, 2010), with a two-tailed statistical significance level set at p < 0.05.

## Results

### Study selection and basic characteristics

**Figure 1:**
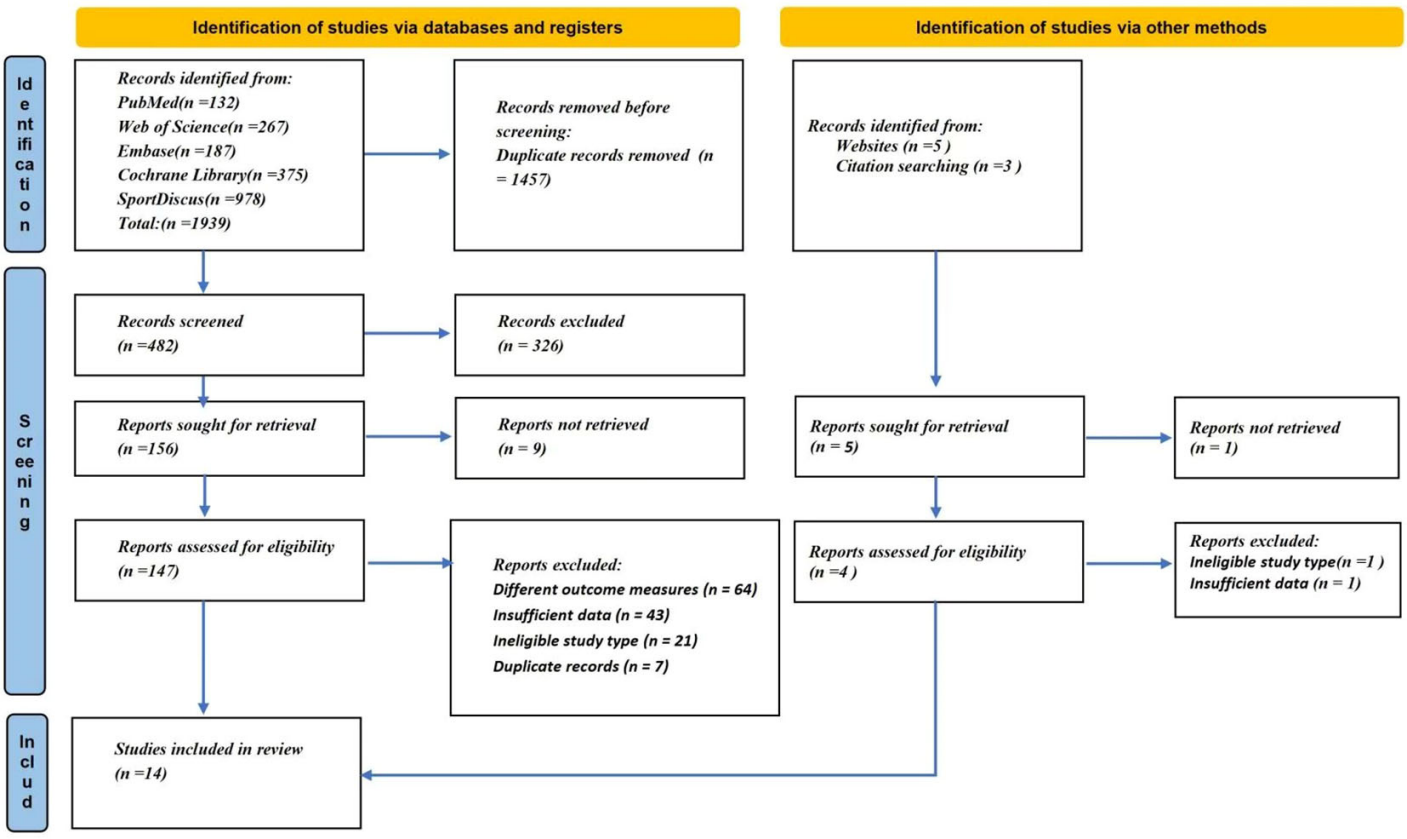
PRISMA 2020 flow diagram illustrating the study selection process from initial database retrieval to final inclusion.

The literature search conducted on March 2, 2026, yielded 1,939 records. Following a preliminary screening, 156 articles were shortlisted for further evaluation. After a full-text review of these studies, 14 fulfilled the eligibility criteria and were incorporated into the meta-analysis.:Aloui et al., 2020; Chaabene et al., 2019; Chelly et al., 2014; de Villareal et al., 2023; Falch et al., 2022; Gaamouri et al., 2023; Hammami et al., 2020a, 2020; Hermassi et al., 2014; Kale et al., 2016; Mazurek et al., 2018; Noutsos et al., 2021; Pancar et al., 2020; Spieszny & Zubik et al., 2018. A detailed flowchart of the study selection process is shown in **Figure 1**.

The final sample of 14 studies comprised a total of 350 participants. Each study adopted a design that compared post-intervention outcomes between an exercise group and a non-intervention control group. The primary endpoints for analysis were performance in the squat jump, countermovement jump and the 10−meter, 20−meter, and 30−meter sprints. The intervention duration varied across the included trials. A summary of the key study characteristics is provided in **Table 1**.

**Table 1:** Characteristics of included literature.

Author	Country	N (total sample)	Measure	Training weeks	% female	Training frequency (per week)	Age (years)	Core exercises	Competitive level
Aloui et al. (2020)	Tunisia	29	30 m sprint (s) countermovement jump (cm) squat jump (cm)	8weeks	0%	2	18.1 ± 0.5	Depth jump, box jump	Junior national male level
Chaabene et al. (2019)	Tunisia	21	countermovement jump (cm)	8weeks	100%	2	15/16	Box jump, continuous double-leg hurdle jumps	Adolescent regional female level
Chelly et al. (2014)	Tunisia	23	countermovement jump (cm) squat jump (cm)	8weeks	0%	3	17.4 ± 0.5	Depth jump, jump shot simulation drills	Elite professional national male level
de Villareal et al. (2023)	Spain	24	countermovement jump (cm)	8weeks	0%	2	19.8 ± 2.2	Depth jump, box jump	Regional adult male level
Falch et al. (2022)	Norway	21	countermovement jump (cm) 10 m sprint (s) 20 m sprint (s) 30 m sprint (s)	8weeks	0%	2	17.1 ± 2.4	Box jump, depth jump	Junior national male level
Gaamouri et al. (2023)	Tunisia	28	countermovement jump (cm) squat jump (cm)	10weeks	100%	2	15.8 ± 0.2	Box jump, defensive block simulation jumps	Elite professional national female level
Hammami et al. (2020a)	Tunisia	34	countermovement jump (cm) squat jump (cm) 10 m sprint (s) 20 m sprint (s) 30 m sprint (s)	10weeks	100%	2	15.8 ± 0.2	Box jump, depth jump	Adolescent national female level
Hammami et al. (2022b)	Tunisia	20	countermovement jump (cm) squat jump (cm) 10 m sprint (s) 20 m sprint (s)	7weeks	0%	3	16.4 ± 0.5	Depth jump, single-leg bounds	Adolescent national male level
Hermassi (2014)	Tunisia	24	countermovement jump(cm) squat jump(cm)	8weeks	0%	3	20 ± 0.3	Depth jump, weighted box jump	Elite professional national male level
Kale (2016)	Turkey	19	countermovement jump (cm) squat jump (cm) 10 m sprint (s) 20 m sprint (s) 30 m sprint (s)	6weeks	100%	2	20.4 ± 3.0	Box jump, depth jump	Regional adult female level
Mazurek (2018)	Poland	26	countermovement jump (cm) squat jump (cm)	5weeks	0%	2	20.2 ± 2.2	Box jump, depth jump	Regional adult male level
Noutsos et al. (2021)	Greece	33	countermovement jump (cm) squat jump (cm) 10 m sprint (s) 20 m sprint (s)	6weeks	0%	2	12.4 ± 2.1	Box jump, depth jump	Pre-adolescent national male level
Pancar (2020)	Turkey	28	30 m sprint (s)	8weeks	100%	2	13.1 ± 0.8	Box jump, depth jump	Pre-adolescent regional female level
Spieszny & Zubik (2018)	Poland	20	countermovement jump (cm) squat jump (cm)	16weeks	0%	2	21.1 ± 2.17	Depth jump, weighted box jump	Regional adult male level

### Risk of bias and publication bias

**Figure 2:**
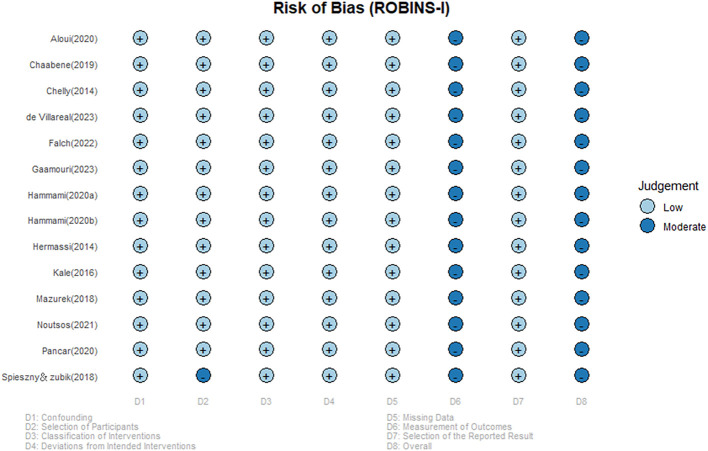
Risk of bias summary assessed by the ROBINS-I tool across seven domains for all 14 included studies.

The overall risk of bias for the included studies, as assessed by the ROBINS-I tool, was predominantly low to moderate (**Figure 2**). Low risk was frequently observed in the domains of confounding (D1), classification of interventions (D3), deviations from intended interventions (D4), missing data (D5), and selection of the reported result (D7). In contrast, biases were more common in the domain of measurement of outcomes (D6), primarily attributable to a lack of assessor blinding, with one study (Spieszny & Zubik, 2018) also showing moderate risk in participant selection (D2) due to non−random allocation. No study was rated as having a serious risk of bias. Therefore, the methodological quality of the evidence base is considered acceptable, and the potential influence of bias on the overall results is likely limited.

### Sensitivity analysis

**Figure 3:**
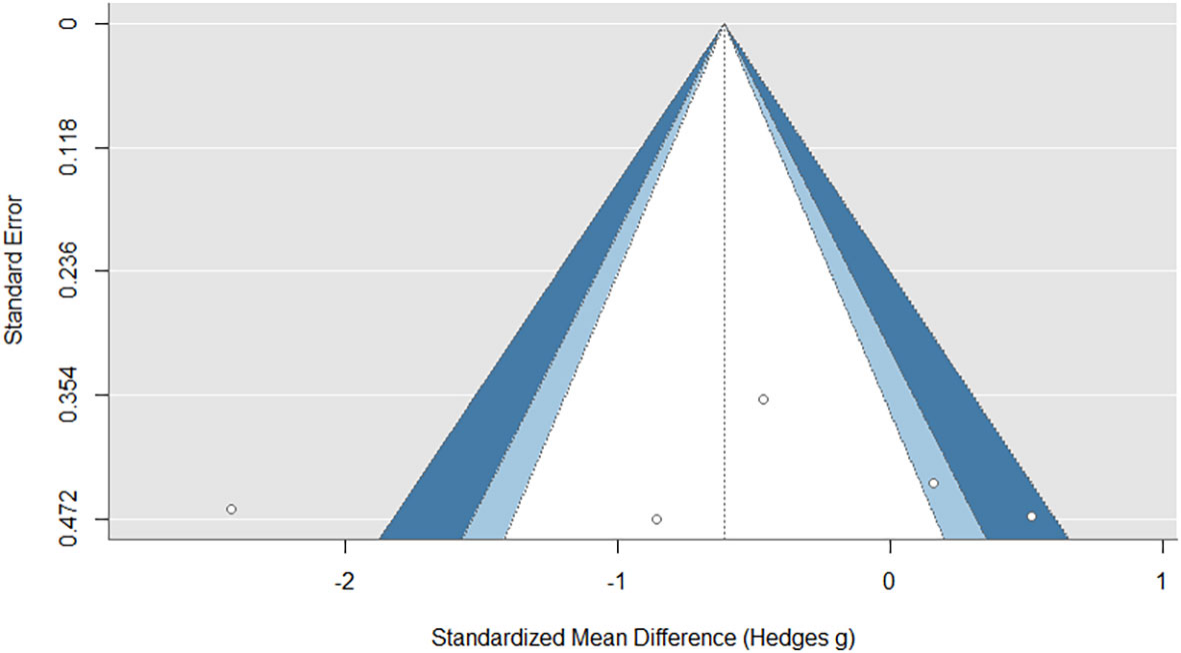
Funnel plot for publication bias assessment of squat jump (SJ) performance, with Hedges’ g on the x-axis and standard error on the y-axis.

For the squat jump (SJ) outcome, the funnel plot exhibited a slight degree of asymmetry, characterized by a trend toward smaller, less precise studies clustering on the right side of the pooled estimate, alongside a relative paucity of studies with null or negative findings on the left (**Figure 3**). This visual suggestion of potential publication bias was marginally supported by statistical testing, as Egger’s regression test yielded a borderline significant result (z = 1.956, p = 0.050). Consequently, while the pooled squat jump estimate should be interpreted with cautious consideration of the observed asymmetry and borderline statistical evidence, the lack of a clearly significant p-value (p = 0.050) also means definitive confirmation of small-study effects or publication bias cannot be asserted.

**Figure 4:**
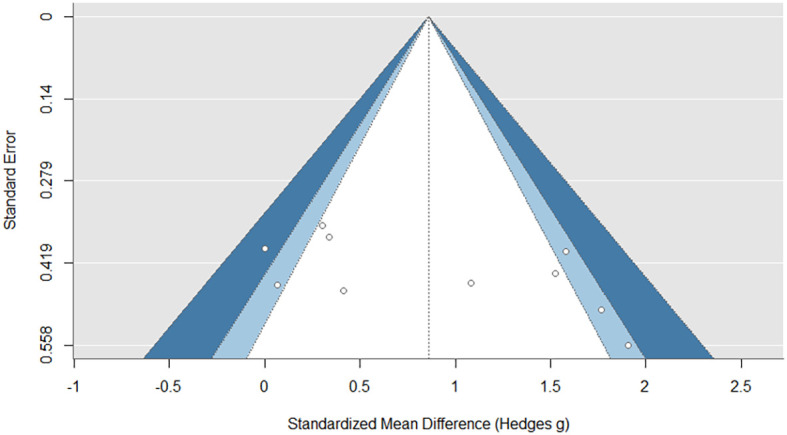
Funnel plot for publication bias assessment of countermovement jump (CMJ) performance, with Hedges’ g on the x-axis and standard error on the y-axis.

For the countermovement jump (CMJ) outcome, the funnel plot exhibited a notable degree of asymmetry, characterized by an aggregation of smaller, less precise studies favoring higher effect sizes on the right, alongside a clear absence of studies with null or negative findings on the left (**Figure 4**). This visual suggestion of publication bias was strongly corroborated by statistical testing, as Egger’s regression test was highly significant (z = 5.597, p = 0.000). Consequently, the pooled countermovement jump estimate should be interpreted with substantial caution, as both visual inspection and statistical evidence indicate definitive small-study effects and publication bias, which may overestimate the true effect of plyometric training on this outcome.

**Figure 5:**
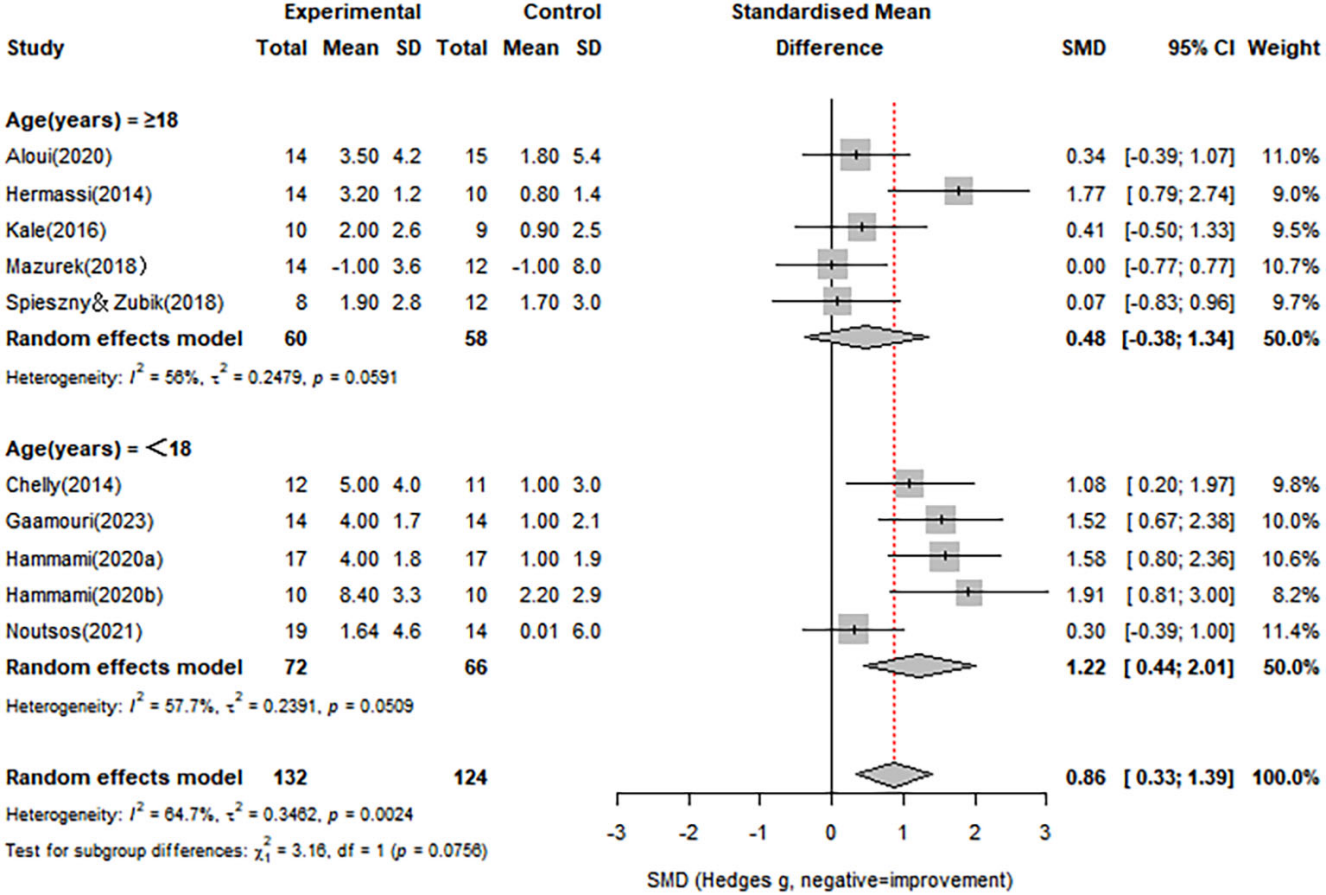
Funnel plot for publication bias assessment of 10 m linear sprint performance, with Hedges’ g on the x-axis and standard error on the y-axis.

For the 10−meter sprint outcome, the funnel plot exhibited a high degree of symmetry, with effect sizes distributed evenly around the pooled estimate (**Figure 5**). No obvious aggregation of smaller, less precise studies favoring larger effect sizes was observed, and there was no notable absence of studies with null or negative findings. This visual suggestion of no publication bias was further corroborated by statistical testing, as Egger’s regression test was non-significant (z = 0.094, p = 0.925). Consequently, the pooled 10−meter sprint estimate can be interpreted with reasonable confidence, as both visual inspection and statistical testing indicate no definitive evidence of small-study effects or publication bias.

**Figure 6:**
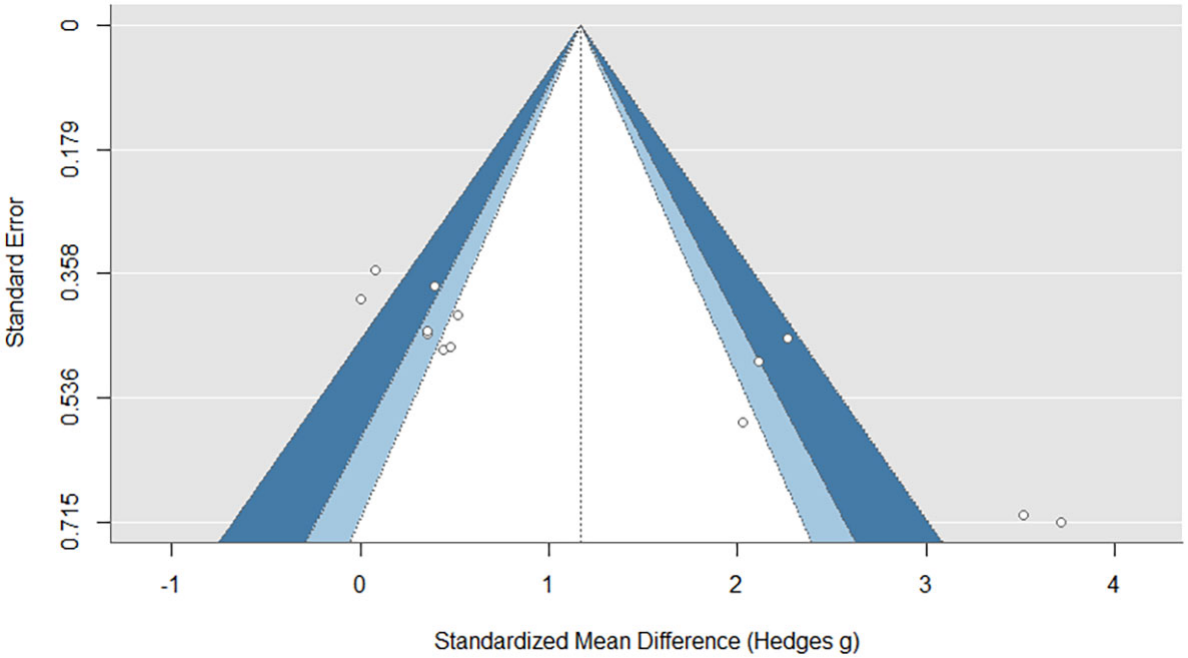
Funnel plot for publication bias assessment of 20 m linear sprint performance, with Hedges’ g on the x-axis and standard error on the y-axis.

For the 20−meter sprint outcome, the funnel plot exhibited a high degree of symmetry, with effect sizes distributed evenly around the pooled estimate (**Figure 6**). No obvious aggregation of smaller, less precise studies favoring larger effect sizes was observed, and there was no notable absence of studies with null or negative findings. This visual suggestion of no publication bias was further corroborated by statistical testing, as Egger’s regression test was non-significant (z = -0.241, p = 0.829). Consequently, the pooled 20−meter sprint estimate can be interpreted with reasonable confidence, as both visual inspection and statistical testing indicate no definitive evidence of small-study effects or publication bias.

**Figure 7:**
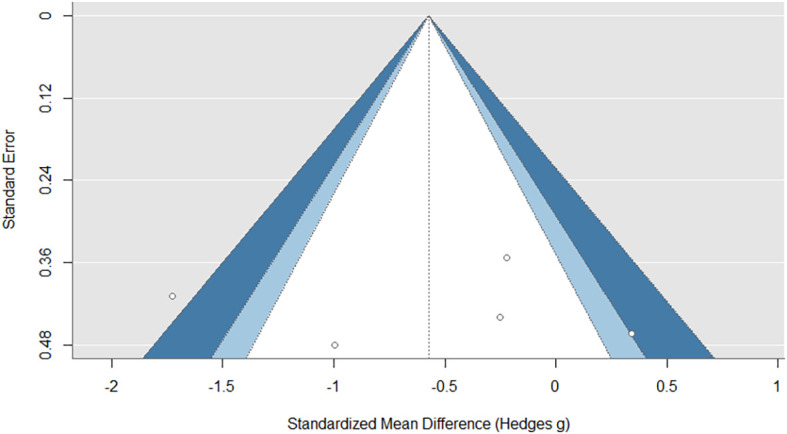
Funnel plot for publication bias assessment of 30 m linear sprint performance, with Hedges’ g on the x-axis and standard error on the y-axis.

For the 30−meter sprint outcome, the funnel plot exhibited a marked degree of asymmetry, characterized by an aggregation of smaller, less precise studies favoring more negative effect sizes on the left, alongside a relative absence of studies with positive or null findings on the right (**Figure 7**). This visual suggestion of publication bias was strongly corroborated by statistical testing, as Egger’s regression test was statistically significant (z = -3.069, p = 0.02). Consequently, the pooled 30−meter sprint estimate should be interpreted with considerable caution, as both visual inspection and statistical evidence confirm the presence of small-study effects and publication bias, which may lead to an overestimation of the true effect of plyometric training on this outcome.

### Main effect results

#### Squat jump

**Figure 8:**
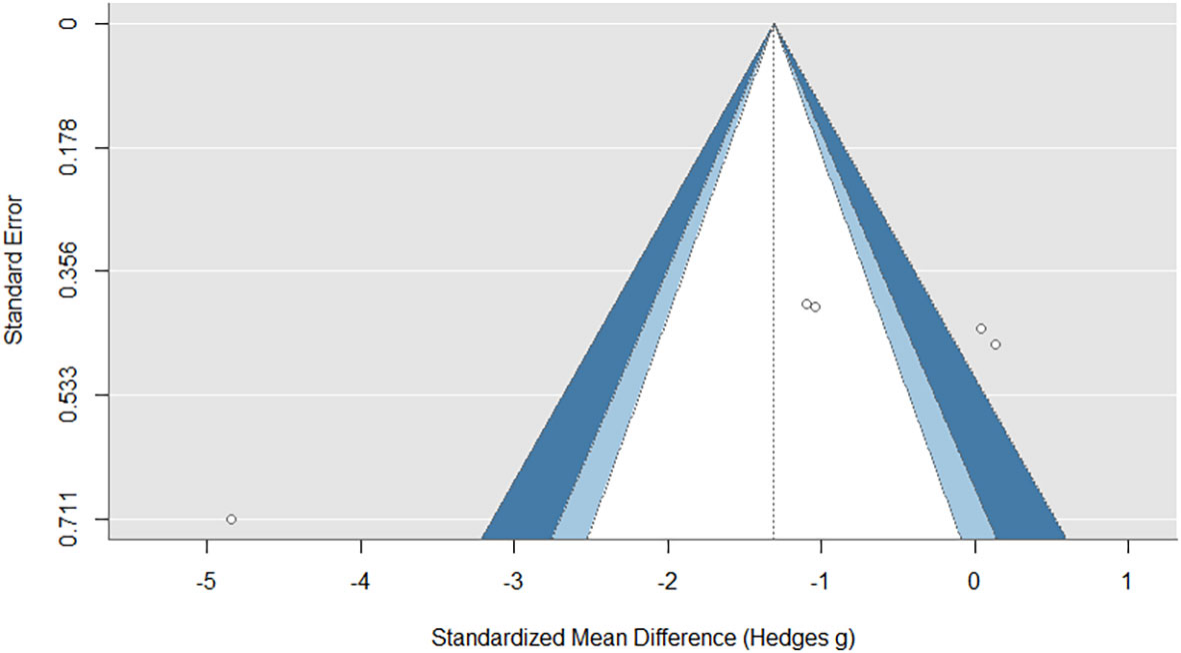
Forest plot of the overall effect of standalone plyometric training on squat jump (SJ) performance using a random-effects model.

For squat jump performance, ten studies with 132 participants in the intervention groups and 124 in the control groups were included. The pooled random-effects analysis revealed a significant overall effect of plyometric training on squat jump performance compared with control conditions (Standardized Mean Difference [SMD] = 0.86, 95% CI 0.33 to 1.39, p = 0.002). Substantial between-study heterogeneity was observed (I² = 64.7%, τ² = 0.3462, p = 0.0024), and the majority of included studies reported favorable effects of plyometric training on squat jump performance, as illustrated in **Figure 8**.

**Figure 9:**
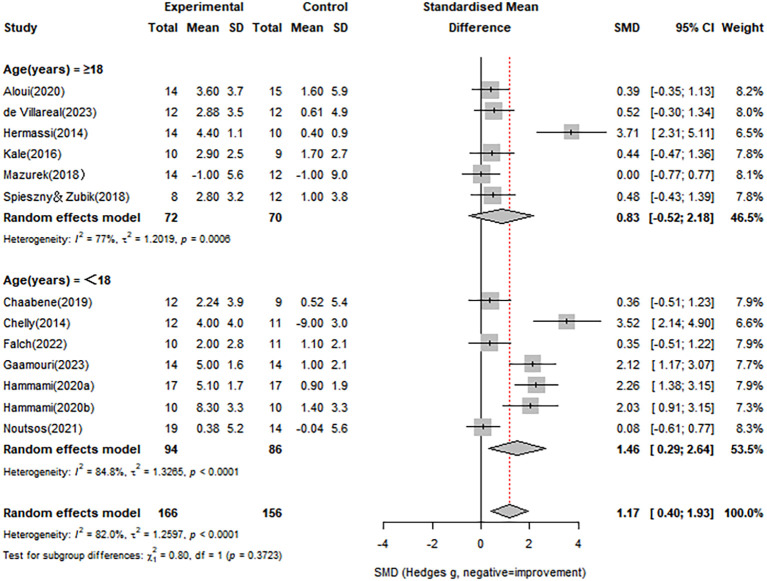
Forest plot of age subgroup analysis for squat jump (SJ) performance, comparing groups aged <18 years and ≥18 years.

To explore the potential influence of age, participants were divided into <18 and ≥18 year old groups. The younger group showed a significant effect relative to controls (Hedges’ g = 1.22, 95% CI 0.44–2.01), while the older group did not (Hedges’ g = 0.48, 95% CI -0.38–1.34), as presented in **Figure 9**.

#### Countermovement jump

**Figure 10:**
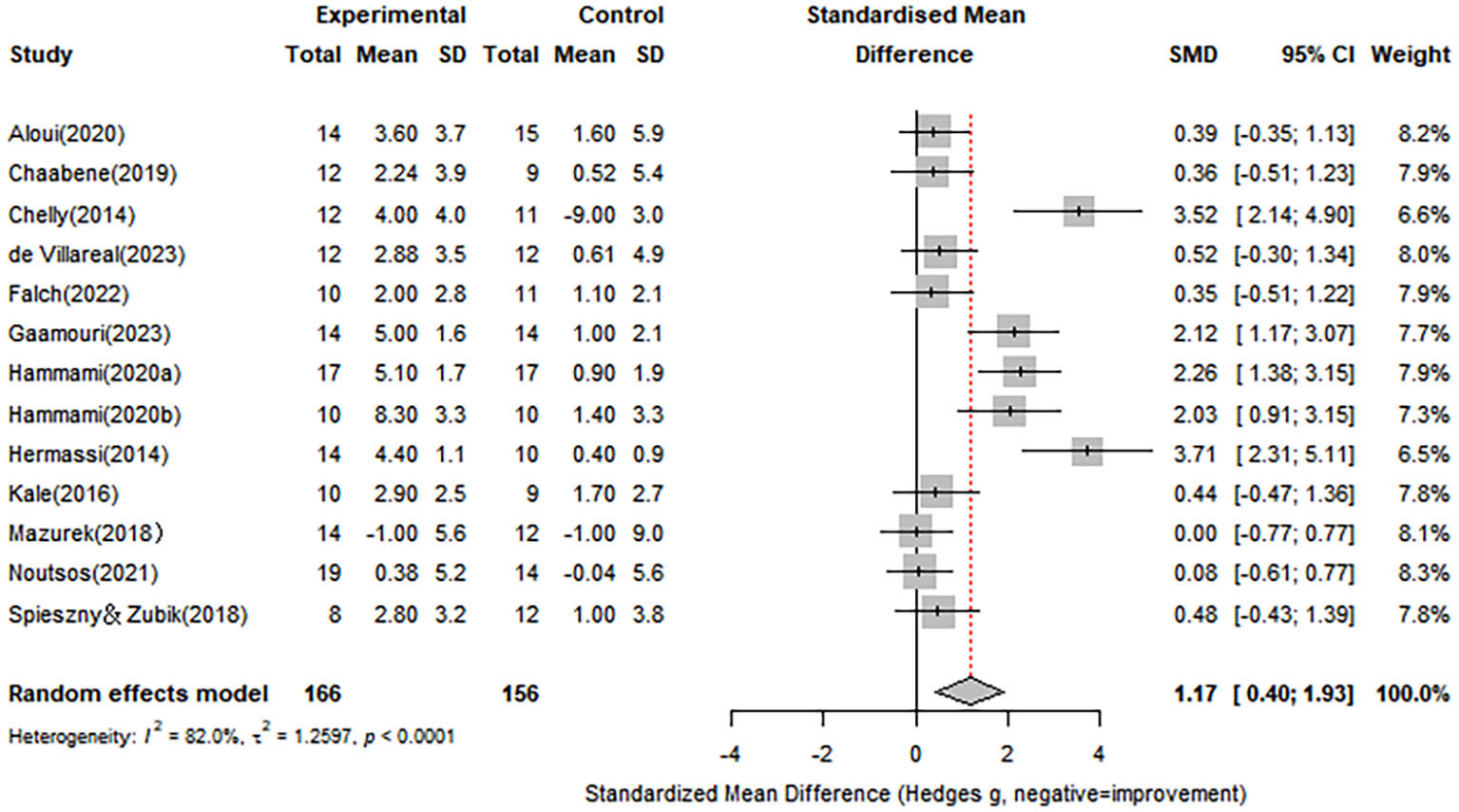
Forest plot of the overall effect of standalone plyometric training on countermovement jump (CMJ) performance using a random-effects model.

For countermovement jump performance, twelve studies with 166 participants in the intervention groups and 156 in the control groups were included. The pooled random-effects analysis revealed a significant overall effect of plyometric training compared with control conditions (Standardized Mean Difference [SMD] = 1.17, 95% CI 0.40 to 1.93, p = 0.004). Very high between-study heterogeneity was observed (I² = 82.0%, τ² = 1.2597, p < 0.0001), and most individual studies demonstrated favorable effects on countermovement jump performance, as detailed in **Figure 10**.

**Figure 11:**
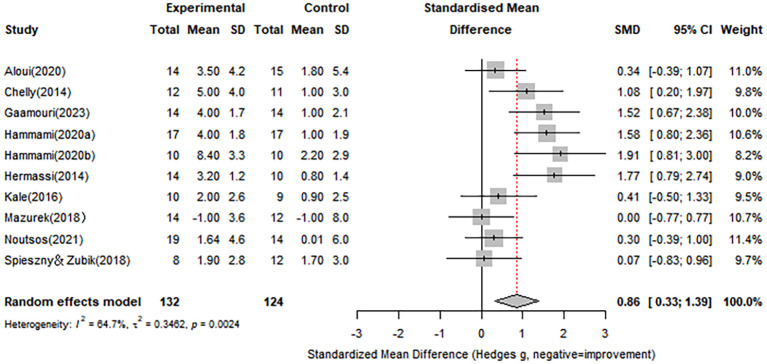
Forest plot of age subgroup analysis for countermovement jump (CMJ) performance, comparing groups aged <18 years and ≥18 years.

To explore the potential influence of age, participants were divided into <18 and ≥18 year old groups. The younger group showed a significant effect relative to controls (Hedges’ g = 1.46, 95% CI 0.29–2.64), while the older group did not (Hedges’ g = 0.83, 95% CI -0.52–2.18), as presented in **Figure 11**.

**Figure 12:**
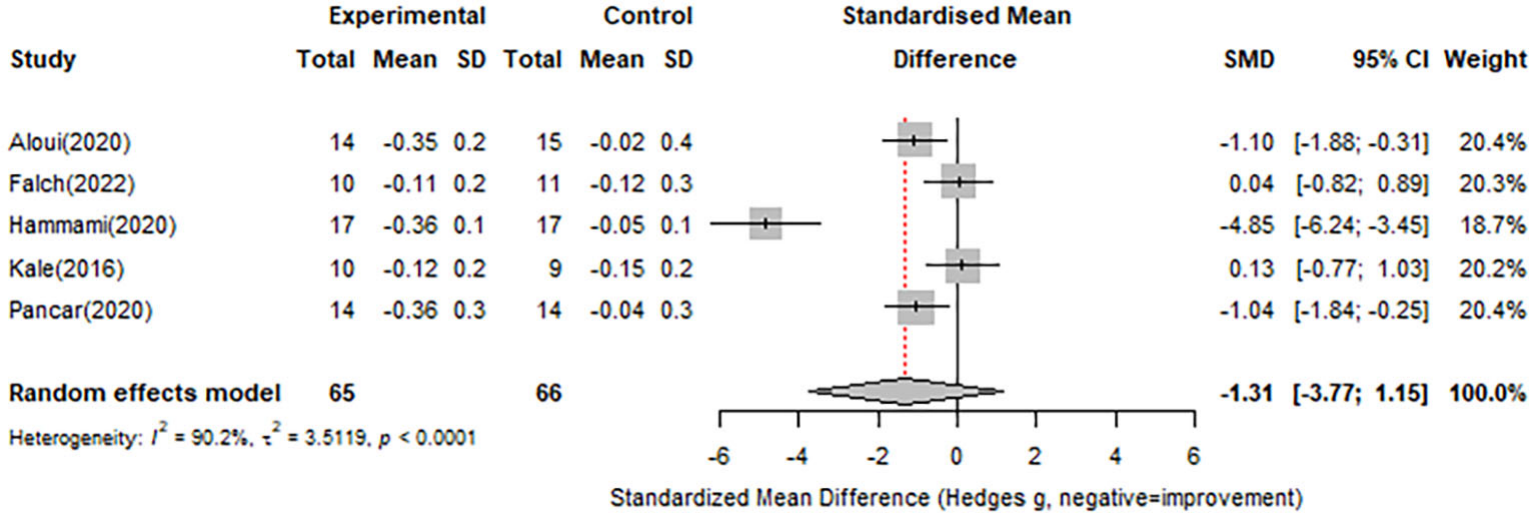
Forest plot of gender subgroup analysis for countermovement jump (CMJ) performance, comparing male and female athletes.

To examine gender differences, a subgroup analysis by gender (male vs. female) was conducted. The male subgroup demonstrated no significant effect (Hedges’ g = 1.25, 95% CI -0.00–2.50), whereas the female subgroup also showed no significant effect (Hedges’ g = 1.10, 95% CI -0.13–2.33); see **Figure 12** for details.

#### 10 m sprint

**Figure 13:**
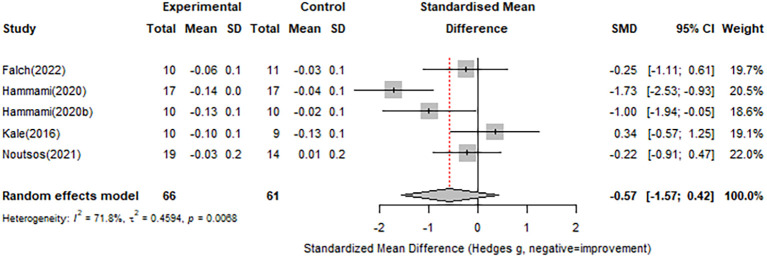
Forest plot of the overall effect of standalone plyometric training on 10 m linear sprint performance using a random-effects model.

For 10 m sprint performance, five studies with 66 participants in the intervention groups and 61 in the control groups were included. The pooled random-effects analysis showed no significant overall effect of plyometric training compared with control conditions (Standardized Mean Difference [SMD] = -0.57, 95% CI -1.57 to 0.42). High between-study heterogeneity was observed (I² = 71.8%, τ² = 0.4594, p = 0.0068), with inconsistent effects reported across individual studies, as detailed in **Figure 13**.

#### 20 m sprint

**Figure 14:**
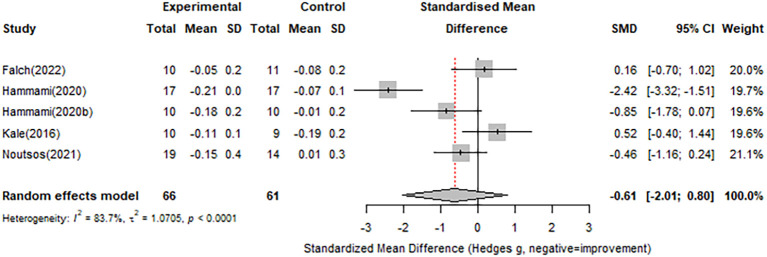
Forest plot of the overall effect of standalone plyometric training on 20 m linear sprint performance using a random-effects model.

For 20 m sprint performance, five studies with 66 participants in the intervention groups and 61 in the control groups were included. The pooled random-effects analysis showed no significant overall effect of plyometric training compared with control conditions (Standardized Mean Difference [SMD] = -0.61, 95% CI -2.01 to 0.80). Very high between-study heterogeneity was observed (I² = 83.7%, τ² = 1.0705, p < 0.0001), with highly inconsistent effects reported across individual studies, as detailed in **Figure 14**.

#### 30 m sprint

**Figure 15:**
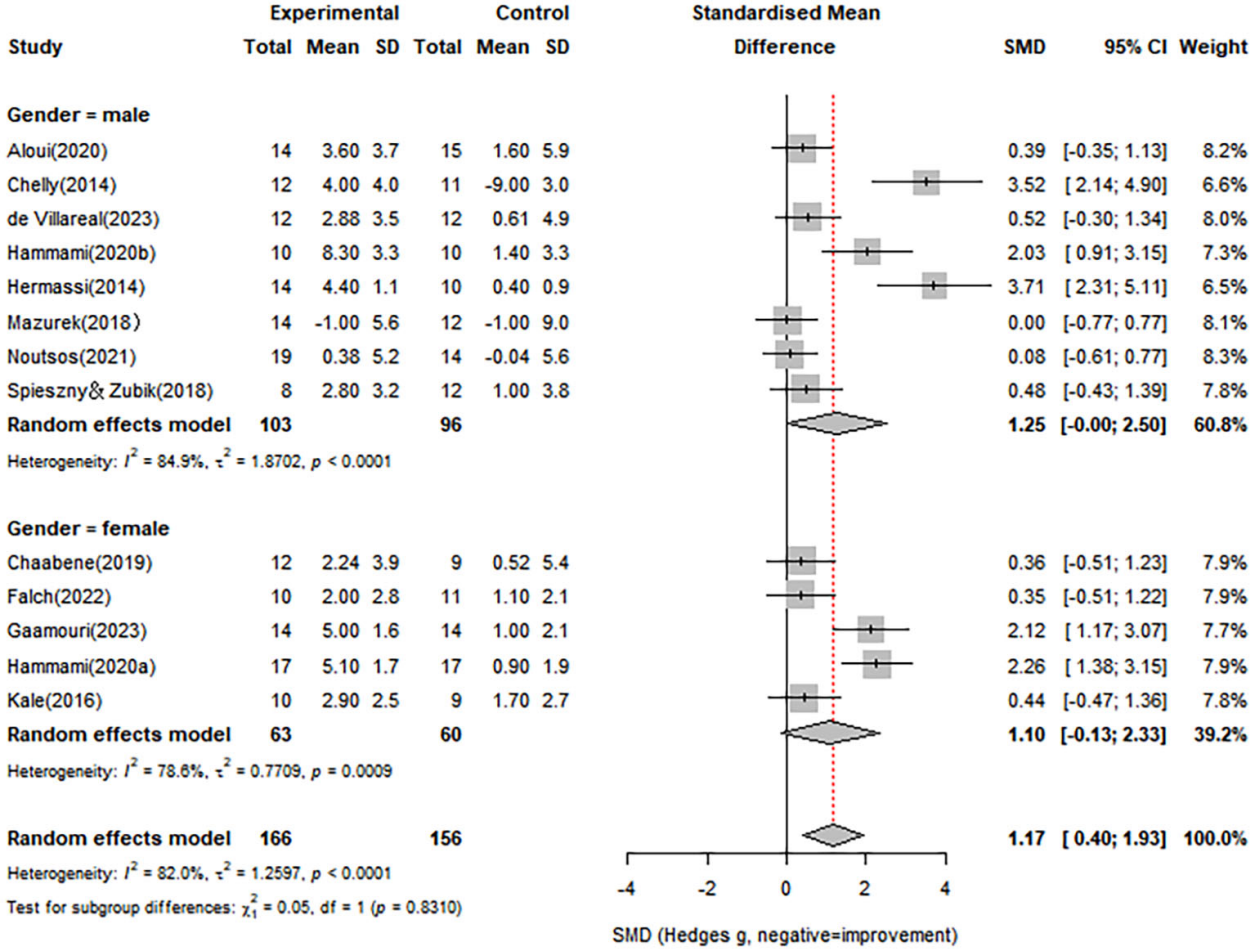
Forest plot of the overall effect of standalone plyometric training on 30 m linear sprint performance using a random-effects model.

For 30 m sprint performance, five studies with 65 participants in the intervention groups and 66 in the control groups were included. The pooled random-effects analysis showed no significant overall effect of plyometric training compared with control conditions (Standardized Mean Difference [SMD] = -1.31, 95% CI -3.77 to 1.15). Very high between-study heterogeneity was observed (I² = 90.2%, τ² = 3.5119, p < 0.0001), with highly inconsistent effects reported across individual studies, as detailed in **Figure 15**.

## Discussion

This systematic review and meta-analysis synthesized existing evidence to evaluate the effects of standalone plyometric training on the physical performance of athletes, specifically focusing on the squat jump, countermovement jump, and the 10-meter, 20-meter, and 30-meter linear sprints. The pooled results demonstrate that plyometric training is an effective method for enhancing lower-body explosive power in this athletic population, although the magnitude of effects, robustness of evidence and presence of potential bias varied substantially across outcomes: statistically significant improvements were observed in both squat jump and countermovement jump performance, confirming the training’s efficacy for vertical jump capabilities, with a larger standardized mean difference for the countermovement jump reflecting a more pronounced effect on this vertical jump measure relative to the squat jump (Kozinc et al., 2023). Notably, the squat jump results exhibited substantial between-study heterogeneity and borderline evidence of publication bias, while the countermovement jump showed very high heterogeneity and definitive publication bias, suggesting training responses for vertical jump performance are highly variable and the observed effects may be overestimated, influenced by small-study effects, individual athlete traits, plyometric program design and study-specific methodological factors. In contrast, the effects of plyometric training on the 10-meter, 20-meter and 30-meter linear sprint performance were all non-significant, with increasingly negative point estimates of effect sizes as sprint distance lengthened, and very high between-study heterogeneity across all sprint measures; the 30-meter sprint additionally presented clear publication bias, while the 10 and 20-meter sprints showed no such bias, collectively indicating a limited and uncertain transfer of plyometric training adaptations to linear sprint capability across short to moderate distances in this athletic population (Suarez-Arrones et al., 2022).

Despite pre-specified subgroup analyses by age and gender, substantial to high between-study heterogeneity persisted across all primary outcomes, with multiple key factors likely contributing to this unexplained variability. First, the included studies showed marked heterogeneity in plyometric program design, with total intervention duration ranging from 5 to 16 weeks, weekly training frequency from 2 to 3 sessions, and large, inconsistently reported differences in total jump volume per session and across the full intervention. As established in existing plyometric literature, training dose is a core moderator of neuromuscular adaptations, and this variability is a primary driver of divergent training responses across studies. Second, training surface conditions were not consistently reported across trials, with interventions conducted on rigid indoor handball courts, sand, or synthetic turf. Previous research has confirmed that training surface directly modulates stretch-shortening cycle (SSC) loading, elastic energy storage and release, and subsequent neuromuscular adaptations, with compliant surfaces like sand associated with reduced impact load and divergent adaptive responses compared to rigid court surfaces. Third, participants’ baseline training status varied widely across studies, from pre-adolescent athletes with limited systematic strength and conditioning experience to elite professional players with multi-year structured training histories. It is well-documented that novice athletes typically exhibit greater plyometric training adaptations than highly trained elite athletes, who are closer to their genetic ceiling for explosive power development, and this variability was not controlled for in our subgroup analyses due to inconsistent reporting across trials. Finally, core exercise selection varied substantially across interventions, including bodyweight versus weighted plyometrics, bilateral versus unilateral jumps, and depth jumps with varying drop heights, each of which targets distinct neuromuscular pathways and elicits different adaptive responses.

### Squat jump

Based on the collective results from multiple studies, the application of plyometric training can be widely recommended as an evidence-based training modality for targeting the specific physical quality underpinning squat jump performance in athletic populations. Notably, the consistent training-induced benefits observed in the studies of Hammami et al. (2020a); Gaamouri et al. (2023) and Hermassi et al. (2014) further validate the robustness of this training method across different athletic cohorts and intervention settings. The overall findings across all included studies provide clear empirical support for the targeted use of plyometric training in strength and conditioning practice for jump performance development. This consistency in training response, observed despite variations in training protocols across studies, suggests that plyometric training exerts a reliable effect on the neuromuscular pathways governing concentric-dominant jump actions, with participant age identified as a critical factor influencing the magnitude of adaptive response: the greater adaptive potential seen in younger athletes highlights the heightened sensitivity of the developing neuromuscular system to plyometric stimuli, consistent with prior meta-analytic evidence documenting age-related gradients in plyometric training adaptations across team sport populations (Ramirez-Campillo et al., 2021; Michailidis et al., 2022; Behm and Chaabene, 2024), while the limited response in adult athletes points to the need for more individualized, periodized intervention strategies for this population, regardless of other potential moderating factors such as training duration or baseline athletic ability (Chaabene et al., 2023). These findings strongly support the core mechanistic rationale for plyometric training, namely that stretch-shortening cycle training drives specific neuromuscular adaptations that directly translate to improved explosive lower-body output, with clear practical implications for strength and conditioning programming in both adolescent and adult athletic populations.

### Countermovement jump

Based on the collective results from multiple studies, plyometric training consistently enhances countermovement jump performance in athletes. Notably, Hermassi et al. (2014), Hammami et al. (2020a) and Gaamouri et al. (2023) reported substantial improvements. The overall findings across all included studies demonstrate a clear positive effect of plyometric training on countermovement jump performance. This consistency in outcomes, observed despite variations in training protocols across studies, suggests that plyometric training effectively improves stretch-shortening cycle (SSC) dependent jump capacity, with participant age emerging as a key moderating factor: further subgroup analysis confirmed that plyometric training delivers significant and consistent benefits to countermovement jump performance in athletes under 18 years of age, while no statistically significant improvement was observed in adult athletes aged 18 and above, whereas no meaningful difference in training response was identified between male and female athletes regardless of other potential moderating factors such as training duration or baseline athletic level (Chaabene et al., 2021; 2025). These findings strongly support the efficacy of plyometric training for developing explosive lower-body power in athletes, particularly adolescent populations, likely through specific neuromuscular and musculoskeletal adaptations including enhanced stretch reflex sensitivity, increased musculotendinous stiffness and optimized motor unit recruitment that directly benefit countermovement jump performance. Caution is warranted in interpreting these findings, however, given the definitive evidence of publication bias identified in the analysis, which may lead to an overestimation of the true training effect.

### Sprint

Based on the collective results from multiple studies, plyometric training does not produce consistent, statistically significant improvements in 10-meter, 20-meter, and 30-meter linear sprint performance in handball athletes. Notably, Aloui et al. (2020), Mazurek et al. (2018) and Spieszny & Zubik (2018) consistently reported non-significant changes in linear sprint performance across all tested distances following standalone plyometric training intervention. The overall findings across all included studies demonstrate that plyometric training has no clear positive effect on short-to-moderate distance linear sprint performance in this athletic population. This consistency in null findings, observed despite variations in training protocols across studies, suggests that the neuromuscular adaptations driven by plyometric training have limited transfer to horizontal sprint movements, regardless of potential moderating factors such as sprint distance or training intervention duration.

Consistent with previous evidence examining the transfer of plyometric training adaptations to linear sprint performance (Lockie et al., 2021), these findings highlight a critical action specificity of plyometric training adaptations, which is further reinforced by the exercise characteristics of the included studies. Among the 14 included trials, the vast majority of interventions were centered on vertical-dominant plyometric exercises (including box jumps, depth jumps, weighted box jumps, and vertical jump shot/defensive block simulation drills), as detailed in **Table 1**. Only 2 of the 14 studies incorporated a small proportion of horizontal-oriented plyometric movements (single-leg bounds), which accounted for less than 20% of the total training volume, and no included study used horizontal-dominant plyometric exercises as the core intervention content. The SSC enhancements induced by vertical-dominant plyometric training are highly specific to vertical force production patterns, and do not readily translate to gains in linear sprint capacity, which relies on distinct horizontal force application, stride mechanics, and SSC utilization patterns. Beyond action specificity, linear sprint performance is a multi-factorial physical quality influenced by step frequency, stride length, technical proficiency, and anterior chain strength, which are not specifically targeted by vertical-dominant plyometric training (Lockie et al., 2023). Caution is warranted when extrapolating the benefits of vertical plyometric training to sprint performance, particularly for longer sprint distances where the risk of effect overestimation due to publication bias is elevated.

## Limitations and future research directions

This meta-analysis has several key limitations. First, significant publication bias was identified for countermovement jump (CMJ, p=0.000) and 30m sprint outcomes, with borderline bias for squat jump (SJ), which may overestimate the true training effect, especially for CMJ. Second, substantial to high between-study heterogeneity persisted across all outcomes after age and gender subgrouping; unexplained variability may stem from differences in training program parameters, exercise surface, athletes’ baseline training status, and core exercise selection. Third, the study was limited by a small overall sample size, few included trials for sprint outcomes, and insufficient statistical power for granular subgroup analyses. Fourth, 7 of the 14 included studies were conducted in Tunisia, creating geographic clustering that may bias results toward the local handball training system and limit the global generalizability of our recommendations. Finally, we did not include handball-specific match performance indicators or long-term follow-up data to assess sustained training benefits.

Future studies should first encourage the publication of null/negative findings to mitigate publication bias. Second, standardized trials with complete reporting of training parameters are needed to reduce heterogeneity and explore additional moderators of training response. Third, large-sample, multi-center studies covering more geographic regions are required to improve the generalizability of findings. Fourth, studies should compare the effects of vertical- versus horizontal-dominant plyometric training on sprint performance to further validate the action specificity argument. Finally, future research should incorporate handball-specific match performance indicators and long-term follow-up, and clarify optimal periodization strategies of standalone plyometric training for handball athletes.

## Conclusion

This meta-analysis presents the first evidence isolating the independent effects of standalone plyometric training on handball athletes’ physical performance, eliminating the confounding of combined training interventions that limited prior research in this field, with three core, non-redundant findings that directly inform on-court conditioning practice. First, standalone plyometric training delivers significant, evidence-based improvements in both SJ and CMJ performance in handball athletes, with the most robust benefits confirmed in athletes under 18 years of age, and no meaningful sex-based difference in training response. Importantly, the highly significant publication bias identified for the CMJ outcome (p=0.000) may lead to an overestimation of the pooled effect size, and the true magnitude of CMJ improvement in practical application may be lower than the pooled Hedges’ g = 1.17 estimate reported in this study; practitioners should interpret this training benefit with appropriate caution. Second, standalone plyometric training yields no significant benefits for 10m, 20m, or 30m linear sprint performance in handball athletes, a finding reinforced by the predominance of vertical-dominant exercises in the included interventions, demonstrating clear action specificity of plyometric training adaptations.

For direct implementation, these findings support a 6–8 week foundational standalone plyometric block delivered 2–3 non-consecutive times weekly for adolescent handball athletes, to drive meaningful improvements in vertical jump performance. For adult handball athletes, adjusted programming integrating individualized external load monitoring and handball-specific jump patterns is required to elicit meaningful neuromuscular adaptations. Notably, standalone vertical-dominant plyometric training should not be used as the primary intervention for linear sprint development in handball athletes. These findings fill a critical gap in handball-specific strength and conditioning evidence, and future research should establish standardized, dose-specific handball plyometric protocols across age, competitive levels, and geographic regions, to refine evidence-based training guidelines for this athletic population.

## Data Availability

The datasets presented in this study can be found in online repositories. The names of the repository/repositories and accession number(s) can be found in the article/**Supplementary Material**.
