# Supplementary material for: Effects of standalone plyometric training on vertical jump and linear sprint performance in handball athletes: a systematic review and meta-analysis
Source: Front Physiol. 2026 Jun 30;17:1863791. doi: 10.3389/fphys.2026.1863791 (PMC13364619; doi:10.3389/fphys.2026.1863791)
Supplement: Supplementary file 2 [file Table2.docx]

**Appendix 1：search strategy**

| **Database** | **Retrieval strategy** |
| --- | --- |
| **Cochranne** | **#1**(plyometric exercise OR plyometric* OR standalone plyometric OR isolated plyometric OR jump training OR explosive training OR stretch shortening cycle) |
|  | #2(handball OR handball player OR handball athletes OR handball players) |
|  | #3 #1 AND #2 |
| **Embase** | **#1**('plyometric exercise'/exp OR plyometric*:ab,ti OR 'standalone plyometric':ab,ti OR 'isolated plyometric':ab,ti OR 'jump training':ab,ti OR 'explosive training':ab,ti OR 'stretch shortening cycle':ab,ti)  #2 ('handball'/exp OR handball*:ab,ti OR 'handball player*':ab,ti OR 'handball athlete*':ab,ti)  #3 #1 AND #2 |
| **Web of Science** | **#1**TS=("plyometric exercise" OR plyometric* OR "standalone plyometric" OR "isolated plyometric" OR "jump training" OR "explosive training" OR "stretch shortening cycle")  #2TS=("handball" OR handball* OR "handball player*" OR "handball athlete*") |
|  | #3 #1 AND #2 |
| **PubMed** | **#1**(plyometric exercise OR plyometric* OR standalone plyometric OR isolated plyometric OR jump training OR explosive training OR stretch shortening cycle)  #2(handball OR handball player OR handball athletes OR handball players)  #3 #1 AND #2 |
| **Sportdiscus** | **#1** (AB(plyometric* OR "standalone plyometric" OR "isolated plyometric" OR "jump training" OR "explosive training" OR "stretch shortening cycle") OR TI(plyometric* OR "standalone plyometric" OR "isolated plyometric" OR "jump training" OR "explosive training" OR "stretch shortening cycle") OR DE "Plyometric Exercise") |
|  | #2(AB(handball* OR "handball player*" OR "handball athlete*") OR TI(handball* OR "handball player*" OR "handball athlete*") OR DE "Handball")  OR DE "Basketball") |
|  | #3 #1 AND #2 |
